# Unusual Presentation of Testicular Cancer with Tumor Thrombus Extending to the Inferior Vena Cava

**DOI:** 10.1155/2015/160560

**Published:** 2015-04-27

**Authors:** Marie Dusaud, Younes Bayoud, François-Régis Desfemmes, Benoît Molimard, Xavier Durand

**Affiliations:** Department of Urology, Military Hospital of Val de Grâce, 74 boulevard de Port Royal, 75005 Paris, France

## Abstract

A 45-year-old man with a left testis tumor with a 25 mm para-aortic lymph node swelling, multiple bilateral pulmonary metastases, bilateral pulmonary embolism, and inferior vena cava (IVC) thrombus underwent a radical orchidectomy in our institution. The thrombus extended from the left gonadal vein to the left renal vein to the IVC. The fluorine-18 fluorodeoxyglucose (f-FDG) positron emission tomography (PET) computerized tomography (CT) demonstrated a hypermetabolic focus in the retroperitoneum and in the IVC thrombus. Before orchidectomy only lactate dehydrogenase (LDH) was high but all the serum tumor markers increased postoperatively. The tumor was staged pT1N2M1aS1, which was an intermediate prognosis, based on the International Germ Cell Cancer Collaborative Group consensus (IGCCCG). After 4 courses of bleomycin, etoposide, and cisplatin (BEP) chemotherapy the patient's tumor markers normalized and the thrombus disappeared. There was only one residual retroperitoneal lymph node M1. Retroperitoneal lymph node dissection was performed. The pathological examination revealed only necrotic tissues. The patient has been disease-free since surgery.

## 1. Introduction

Tumor thrombus is a rare complication of testis cancer. Extension into the inferior vena cava (IVC) and/or the right atrium needs appropriate treatment including chemotherapy and surgery. We report a case of testicular cancer with inferior vena cava tumor thrombus and multiple metastases and we discuss the different options of management after diagnosis.

## 2. Case Presentation

**Figure 1:**
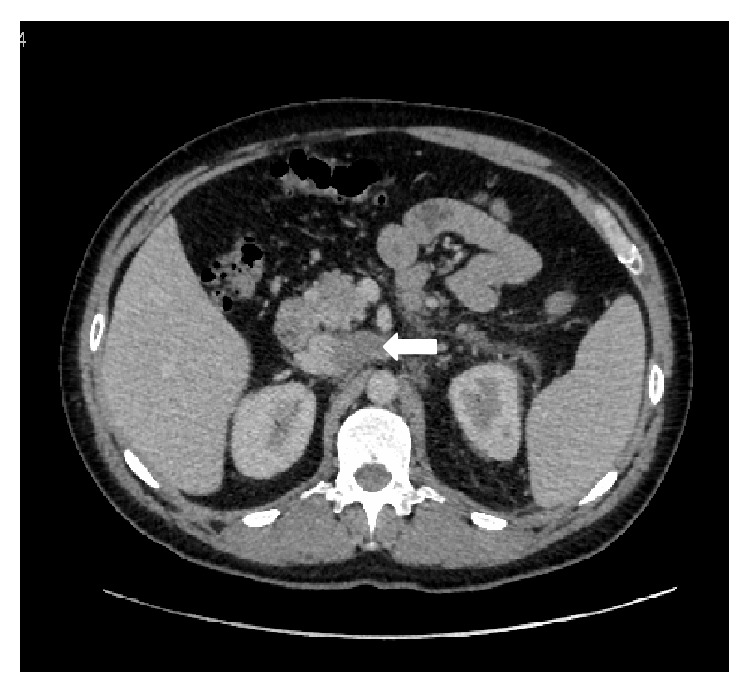
Computerized tomography (CT) scan of the abdomen demonstrating the thrombus extended from the left renal vein to the IVC. The arrow indicates the 40 mm thrombus.

A 45-year-old man presented with a 3-month history of left scrotal pain initially diagnosed as an epididymitis. Physical examination and scrotal US revealed a left testis tumor. Computerized tomography (CT) scan of the chest, abdomen, and pelvis demonstrated a 25 mm para-aortic lymph node swelling, multiple bilateral pulmonary metastases, bilateral pulmonary embolism, and inferior vena cava (IVC) thrombus (Figure 1). The thrombus extended from the left gonadal vein to the left renal vein to the IVC. There was no evidence of collateral development.

**Figure 2:**
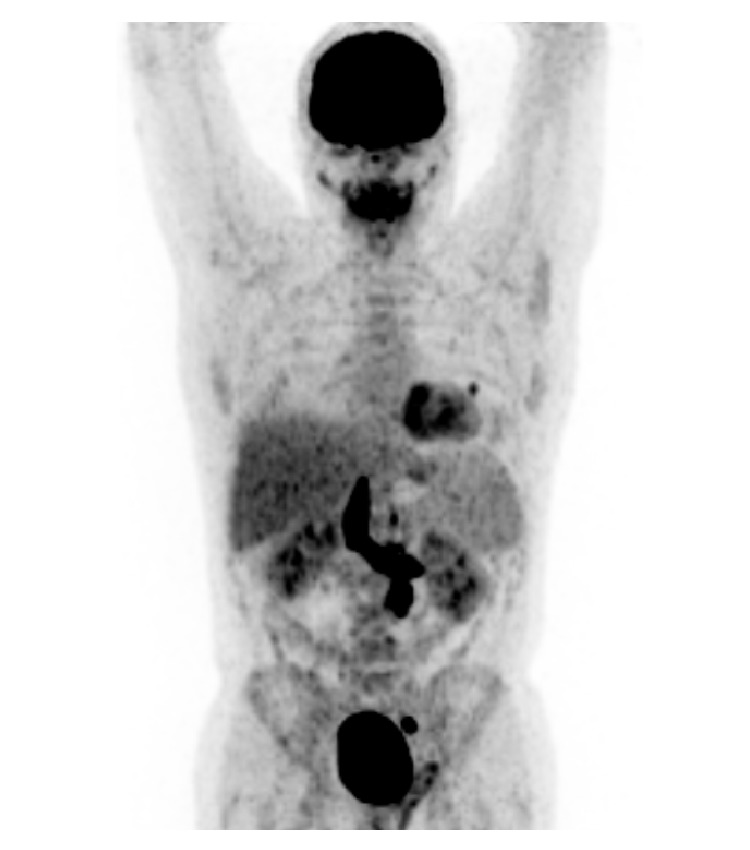
Fluorine-18 fluorodeoxyglucose (f-FDG) positron emission tomography (PET) computerized tomography (CT) showing hypermetabolic IVC thrombus.

We performed a fluorine-18 fluorodeoxyglucose (f-FDG) positron emission tomography (PET) computerized tomography (CT) in order to characterize the thrombus (Figure 2). It demonstrated a hypermetabolic focus in the retroperitoneum (SUV 11,7) and in the IVC thrombus (SUV between 11,7 and 16,6) for 9,5 cm long.

Serum tumor markers were normal except lactate dehydrogenase (LDH): alpha-fetoprotein (AFP) 5,2 *μ*g/L (≤7), human chorionic gonadotrophin (HCG) 2,9 UI/L (≤5), and LDH 440 UI/L (1,8 N).

The patient underwent left inguinal orchidectomy and pathological examination revealed embryonal carcinoma with intratubular germ cell neoplasia (ITGCN). The rete testis was involved by tumor. The epididymitis, the tunica albuginea, and the spermatic cord were free of tumor.

Postoperatively, serum tumor makers were increasing: AFP, HCG, and LDH, respectively, from 5,2 *μ*g/L, 2,9 UI/L, and 440 UI/L to 9,3 *μ*g/L, 14,8 UI/L, and 486 UI/L.

The tumor was diagnosed as a nonseminomatous germ cell tumor (NSCGT) with a clinical stage of pT1N2M1aS1, which was an intermediate prognosis, based on the International Germ Cell Cancer Collaborative Group consensus (IGCCCG).

Chemotherapy was started with the bleomycin, etoposide, and cisplatin (BEP) regimen for four courses and therapeutic anticoagulation was started. Placement of an IVC filter was impossible because the upper limit of the thrombus was too close to the hepatic vein.

After 4 courses of chemotherapy the patient's tumor markers normalized and the thrombus disappeared. There was only one residual retroperitoneal para-aortic lymph node on the CT scan whitch was hypermetabolic on the 18 fDG PET CT.

Retroperitoneal lymph node dissection was performed. The pathological examination revealed only necrotic tissues. The patient has been disease-free at 8 months since surgery.

## 3. Discussion

Involvement of the inferior vena cava (IVC) by a testicular tumor is a rare event. Two autopsy series of patients with testicular germ cell tumors have suggested IVC involvement in 3% and 11% of patients [1, 2]. Husband and Bellamy reviewed the CT scans of 650 patients with testicular cancer and found only 4 cases of IVC invasion among 397 patients with retroperitoneal disease [3].

The diagnosis of IVC thrombus can be performed by CT scan and by magnetic resonance imaging (MRI) [4]. The 18F-FDG-PET/CT can be useful, mainly to prevent unnecessary long-term anticoagulation treatment [5] because it can differentiate nonhypermetabolic bland thrombosis from hypermetabolic tumoral thrombosis that does not require long-term anticoagulation.

There are two mechanisms by which the IVC may be involved. The first is due to the spread of the tumor by direct invasion of the spermatic vein and then of the vena cava. That explains why IVC invasion more frequently occurs with right side tumors because of the direct insertion of the right gonadal vein into the IVC. The second explanation is lymphatic spread and direct invasion from paracaval metastatic sites secondary to the development of lymphatic-venous shunting in severe lymphatic disease. Thus, bulky retroperitoneal disease is a major risk factor for IVC tumor thrombus.

The most significant potential complication of IVC thrombosis is the risk of pulmonary (tumoral) embolism which may lead to death. Bredael et al. reported that 9% of testicular cancer patients died of pulmonary embolism in autopsy cases [1]. Despite heparinization, tumoral pulmonary embolism may occur as described by O'Brien and Lynch [6]. To prevent the risk of pulmonary embolism, Masui et al. decided to insert a temporary IVC filter prior to orchidectomy and chemotherapy [7] with a good result of chemotherapy and no complication.

When the tumoral thrombus is extending to the heart such as the case report by Savarese et al., the risk of embolism is very high [8]. There is an indication of urgent thrombectomy before beginning chemotherapy, which would be delayed by laparotomy and intra-abdominal dissection. They performed a tumor thrombectomy using cardiopulmonary bypass (CBP) and hypothermic circulatory arrest followed 6 days later by chemotherapy without any postoperative complication. In order to avoid operative morbidity due to CBP, Kinebuchi et al. performed thrombectomy using a venovenous bypass (VVB) with success [9].

For patients with an IVC thrombus without cardiac involvement, surgery could be performed after chemotherapy with or without vena caval filter. While reviewing the literature over the past 30 years, we find only 2 cases reporting involvement of the IVC by testicular neoplasm treated by chemotherapy and anticoagulation without any vena cava resection [10, 11] and only one with a complete regression of the thrombus.

The histopathological composition of intraluminal vena caval thrombosis reflects the pathological features present in the testis, most frequently embryonal carcinoma component (43,5%) [9], or is downstaged in fibrosis and/or in teratoma [12].

Testis cancer with an IVC tumor thrombus is a rare event. Aggressive surgical approach before or after chemotherapy is the best therapeutic option for such patients. Most tumor thrombus rarely regresses totally after chemotherapy and thrombus surgical resection is commonly deployed.
